# 2-[5-Bromo-1-(3-chloro­benz­yl)-2-methyl-1*H*-indol-3-yl]acetic acid

**DOI:** 10.1107/S1600536812046740

**Published:** 2012-11-24

**Authors:** Mahmoud Elkady, Peter R. W. E. F. Keck, Dieter Schollmeyer, Stefan Laufer

**Affiliations:** a Eberhard-Karls-University Tübingen, Auf der Morgenstelle 8, 72076 Tübingen, Germany; b University Mainz, Institut of Organic Chemistry, Duesbergweg 10-14, 55099 Mainz, Germany

## Abstract

In the title compound, C_18_H_15_BrClNO_2_, the indole ring system forms a dihedral angle of 86.9 (2)° with the 3-chloro­benzyl ring. In the crystal, mol­ecules form inversion dimers connected *via* pairs of O—H⋯O hydrogen bonds.

## Related literature

For biological activity of inhibitors for the microsomal prostaglandin E_2_ synthase-1 (mPGES-1) and 5-lipoxygenase (5-LO), see: Elkady *et al.* (2012 ▶). For details of the synthesis, see: Maguire *et al.* (2001 ▶).
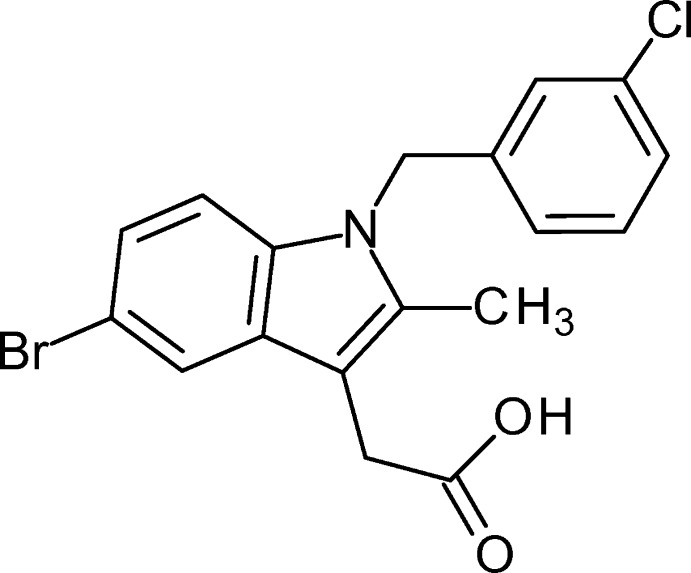


## Experimental

### 

#### Crystal data

C_18_H_15_BrClNO_2_
*M* *_r_* = 392.67
Triclinic, 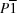

*a* = 8.5386 (11) Å
*b* = 10.0157 (10) Å
*c* = 11.0821 (12) Å
α = 109.221 (8)°
β = 106.229 (9)°
γ = 101.886 (9)°
*V* = 811.52 (16) Å^3^
*Z* = 2
Mo *K*α radiation
μ = 2.71 mm^−1^
*T* = 298 K
0.44 × 0.15 × 0.12 mm

#### Data collection

Stoe IPDS 2T diffractometer
Absorption correction: integration (*X-RED*; Stoe & Cie, 2010 ▶) *T*_min_ = 0.388, *T*_max_ = 0.785
9139 measured reflections
4400 independent reflections
2791 reflections with *I* > 2σ(*I*)
*R*_int_ = 0.040

#### Refinement

*R*[*F* ^2^ > 2σ(*F* ^2^)] = 0.052
*wR*(*F* ^2^) = 0.150
*S* = 1.02
4400 reflections
209 parameters
H-atom parameters constrained
Δρ_max_ = 0.72 e Å^−3^
Δρ_min_ = −0.69 e Å^−3^

### 

Data collection: *X-AREA* (Stoe & Cie, 2010 ▶); cell refinement: *X-AREA*; data reduction: *X-RED* (Stoe & Cie, 2010 ▶); program(s) used to solve structure: *SIR97* (Altomare *et al.*, 1999 ▶); program(s) used to refine structure: *SHELXL97* (Sheldrick, 2008 ▶); molecular graphics: *PLATON* (Spek, 2009 ▶); software used to prepare material for publication: *PLATON*.

## Supplementary Material

Crystal structure: contains datablock(s) I, global. DOI: 10.1107/S1600536812046740/bt6855sup1.cif

Structure factors: contains datablock(s) I. DOI: 10.1107/S1600536812046740/bt6855Isup2.hkl

Supplementary material file. DOI: 10.1107/S1600536812046740/bt6855Isup3.cml

Additional supplementary materials:  crystallographic information; 3D view; checkCIF report

## Figures and Tables

**Table 1:** Hydrogen-bond geometry (Å, °)

*D*—H⋯*A*	*D*—H	H⋯*A*	*D*⋯*A*	*D*—H⋯*A*
O18—H18⋯O17^i^	0.82	1.87	2.679 (3)	170

Symmetry code: (i) 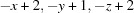.

**Fig. 1.**
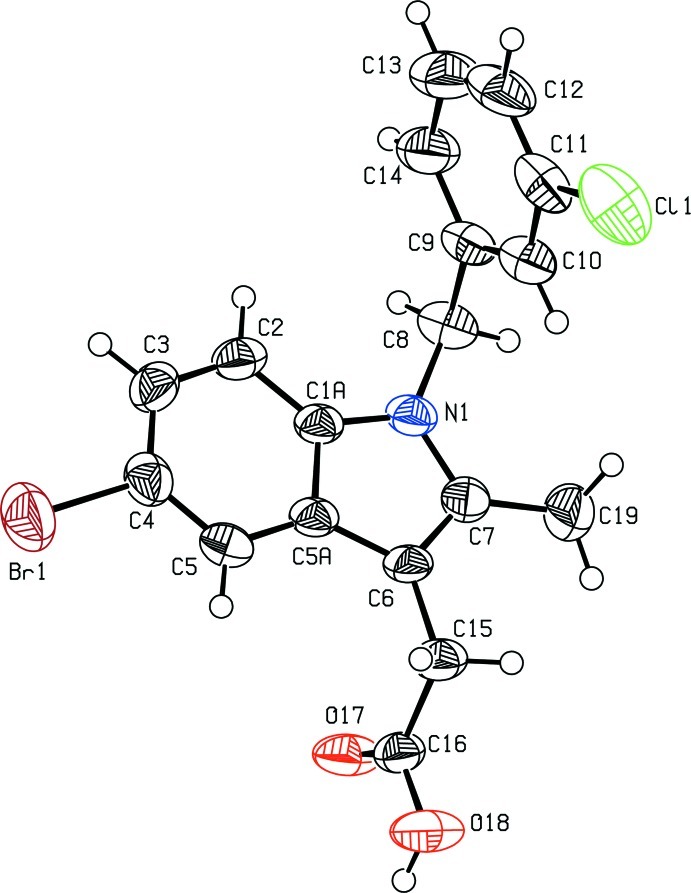
View of compound I. Displacement ellipsoids are drawn at the 50% probability level.

## supplementary crystallographic information

### Comment

We synthesized and evaluated inhibitors for the microsomal prostaglandin E_2_ synthase-1 (mPGES-1) and 5-lipoxygenase (5-LO) (Elkady *et al.*, 2012). The title compound was synthesized to obtain a template which leads to series of different derivates of the indole scaffold.

The indole ring is oriented with dihedral angle of 86.9 (2)° with the 3-chlorobenzyl ring in the crystal structure. Each two molecules form centrosymmetrical dimers connected *via* O18–H18···O17 hydrogen bridges (O18···O17 2.679 (3) Å) (Tab.1).

### Experimental

0.2 g (0.74 mmol) of 2-(5-bromo-2-methyl-1*H*-indol-3-yl) acetic acid were dissolved in 5 ml dry dimethylformamide. Then 60 mg of Sodium hydride were slowly added and the mixture was stirred under argon at 273 K for 30 min (Maguire *et al.*, 2001). Then, 0.18 ml (1.48 mmol) of 3-chlorobenzyl chloride were dissolved in 3 ml of dry dimethylformamide and this was then slowly added to the mixture and stirred for 1 h at 273 K. The reaction was stopped by quenching with ice cooled water; the pH was adjusted to 1 by 5 N aqueous hydrochloric acid. The product was extracted with ethyl acetate three times, dried over anhydrous sodium sulfate and finally concentrated under vacuum. The product was purified by washing with methanol. Crystals of the title compound were obtained by slow evaporation of methanol at room temperature.

### Refinement

Hydrogen atoms were placed at calculated positions with O—H = 0.82 Å, C—H = 0.95 Å (aromatic) or 0.99–1.00 Å (*sp*^3^ C-atom). All H atoms were refined with isotropic displacement parameters set to 1.2–1.5 times of the *U*_eq_ of the parent atom.

### Crystal data

**Table:**

C_18_H_15_BrClNO_2_	*Z* = 2
*M**_r_* = 392.67	*F*(000) = 396
Triclinic, *P*1	*D*_x_ = 1.607 Mg m^−^^3^
Hall symbol: -P 1	Mo *K*α radiation, λ = 0.71073 Å
*a* = 8.5386 (11) Å	Cell parameters from 6836 reflections
*b* = 10.0157 (10) Å	θ = 2.4–29.3°
*c* = 11.0821 (12) Å	µ = 2.71 mm^−^^1^
α = 109.221 (8)°	*T* = 298 K
β = 106.229 (9)°	Block, colourless
γ = 101.886 (9)°	0.44 × 0.15 × 0.12 mm
*V* = 811.52 (16) Å^3^	

### Data collection

**Table:**

Stoe IPDS 2T diffractometer	4400 independent reflections
Radiation source: sealed Tube	2791 reflections with *I* > 2σ(*I*)
Graphite monochromator	*R*_int_ = 0.040
Detector resolution: 6.67 pixels mm^-1^	θ_max_ = 29.3°, θ_min_ = 2.4°
rotation method scans	*h* = −11→11
Absorption correction: integration (*X-RED*; Stoe & Cie, 2010)	*k* = −13→12
*T*_min_ = 0.388, *T*_max_ = 0.785	*l* = −15→15
9139 measured reflections	

### Refinement

**Table:**

Refinement on *F*^2^	Primary atom site location: structure-invariant direct methods
Least-squares matrix: full	Secondary atom site location: difference Fourier map
*R*[*F*^2^ > 2σ(*F*^2^)] = 0.052	Hydrogen site location: inferred from neighbouring sites
*wR*(*F*^2^) = 0.150	H-atom parameters constrained
*S* = 1.02	*w* = 1/[σ^2^(*F*_o_^2^) + (0.069*P*)^2^ + 0.4677*P*] where *P* = (*F*_o_^2^ + 2*F*_c_^2^)/3
4400 reflections	(Δ/σ)_max_ < 0.001
209 parameters	Δρ_max_ = 0.72 e Å^−^^3^
0 restraints	Δρ_min_ = −0.69 e Å^−^^3^

### Special details

**Table:**

Geometry. All e.s.d.'s (except the e.s.d. in the dihedral angle between two l.s. planes) are estimated using the full covariance matrix. The cell e.s.d.'s are taken into account individually in the estimation of e.s.d.'s in distances, angles and torsion angles; correlations between e.s.d.'s in cell parameters are only used when they are defined by crystal symmetry. An approximate (isotropic) treatment of cell e.s.d.'s is used for estimating e.s.d.'s involving l.s. planes.
Refinement. Refinement of *F*^2^ against ALL reflections. The weighted *R*-factor *wR* and goodness of fit *S* are based on *F*^2^, conventional *R*-factors *R* are based on *F*, with *F* set to zero for negative *F*^2^. The threshold expression of *F*^2^ > σ(*F*^2^) is used only for calculating *R*-factors(gt) *etc*. and is not relevant to the choice of reflections for refinement. *R*-factors based on *F*^2^ are statistically about twice as large as those based on *F*, and *R*- factors based on ALL data will be even larger.

### Fractional atomic coordinates and isotropic or equivalent isotropic displacement parameters (Å^2^)

**Table:**

	*x*	*y*	*z*	*U*_iso_*/*U*_eq_	
Br1	0.98960 (7)	0.99173 (4)	0.68263 (5)	0.0811 (2)	
Cl1	0.82521 (16)	0.14242 (15)	−0.02826 (13)	0.0943 (4)	
N1	0.5804 (3)	0.3419 (3)	0.3758 (2)	0.0436 (5)	
C1A	0.6557 (4)	0.4936 (3)	0.4301 (3)	0.0398 (6)	
C2	0.6079 (4)	0.5993 (4)	0.3871 (3)	0.0478 (7)	
H2	0.5097	0.5714	0.3094	0.057*	
C3	0.7091 (5)	0.7457 (4)	0.4622 (3)	0.0520 (8)	
H3	0.6801	0.8188	0.4351	0.062*	
C4	0.8553 (5)	0.7865 (4)	0.5788 (3)	0.0516 (7)	
C5	0.9056 (4)	0.6835 (4)	0.6232 (3)	0.0465 (7)	
H5	1.0037	0.7130	0.7015	0.056*	
C5A	0.8048 (4)	0.5337 (3)	0.5472 (3)	0.0396 (6)	
C6	0.8162 (4)	0.3989 (3)	0.5604 (3)	0.0426 (6)	
C7	0.6776 (4)	0.2843 (3)	0.4540 (3)	0.0458 (7)	
C8	0.4319 (4)	0.2570 (4)	0.2463 (3)	0.0527 (7)	
H8A	0.3432	0.3038	0.2471	0.063*	
H8B	0.3848	0.1564	0.2387	0.063*	
C9	0.4776 (4)	0.2482 (4)	0.1228 (3)	0.0476 (7)	
C10	0.6134 (4)	0.2012 (4)	0.1056 (3)	0.0521 (7)	
H10	0.6778	0.1724	0.1692	0.063*	
C11	0.6520 (5)	0.1976 (4)	−0.0080 (3)	0.0602 (9)	
C12	0.5554 (6)	0.2388 (5)	−0.1038 (3)	0.0733 (12)	
H12	0.5833	0.2366	−0.1797	0.088*	
C13	0.4217 (6)	0.2818 (5)	−0.0869 (4)	0.0765 (12)	
H13	0.3560	0.3083	−0.1519	0.092*	
C14	0.3804 (5)	0.2871 (5)	0.0259 (3)	0.0644 (9)	
H14	0.2871	0.3169	0.0367	0.077*	
C15	0.9604 (5)	0.3856 (4)	0.6627 (3)	0.0514 (7)	
H15A	1.0681	0.4430	0.6646	0.062*	
H15B	0.9548	0.2816	0.6302	0.062*	
C16	0.9669 (4)	0.4356 (3)	0.8074 (3)	0.0436 (6)	
O17	0.8593 (3)	0.4791 (3)	0.8448 (2)	0.0632 (7)	
O18	1.1017 (3)	0.4268 (4)	0.8893 (2)	0.0717 (8)	
H18	1.1012	0.4549	0.9675	0.108*	
C19	0.6253 (6)	0.1216 (4)	0.4211 (4)	0.0663 (10)	
H19A	0.7000	0.1043	0.4928	0.099*	
H19B	0.6339	0.0682	0.3349	0.099*	
H19C	0.5083	0.0871	0.4141	0.099*	

### Atomic displacement parameters (Å^2^)

**Table:**

	*U*^11^	*U*^22^	*U*^33^	*U*^12^	*U*^13^	*U*^23^
Br1	0.1083 (4)	0.0471 (2)	0.0743 (3)	0.0105 (2)	0.0368 (3)	0.01569 (18)
Cl1	0.0830 (8)	0.0973 (8)	0.0888 (8)	0.0194 (6)	0.0543 (7)	0.0096 (6)
N1	0.0509 (14)	0.0486 (13)	0.0299 (10)	0.0130 (11)	0.0149 (10)	0.0167 (10)
C1A	0.0468 (15)	0.0489 (15)	0.0299 (11)	0.0190 (12)	0.0183 (11)	0.0185 (11)
C2	0.0545 (18)	0.0626 (18)	0.0394 (14)	0.0274 (15)	0.0207 (13)	0.0289 (14)
C3	0.071 (2)	0.0532 (17)	0.0516 (17)	0.0312 (16)	0.0327 (17)	0.0310 (15)
C4	0.067 (2)	0.0444 (15)	0.0469 (16)	0.0165 (14)	0.0307 (15)	0.0172 (13)
C5	0.0494 (17)	0.0543 (17)	0.0339 (13)	0.0152 (14)	0.0171 (12)	0.0160 (12)
C5A	0.0476 (16)	0.0494 (15)	0.0287 (11)	0.0198 (12)	0.0188 (11)	0.0184 (11)
C6	0.0575 (18)	0.0530 (16)	0.0280 (12)	0.0266 (14)	0.0198 (12)	0.0213 (11)
C7	0.0638 (19)	0.0467 (15)	0.0363 (13)	0.0209 (14)	0.0264 (13)	0.0201 (12)
C8	0.0466 (17)	0.0630 (19)	0.0382 (14)	0.0075 (15)	0.0134 (13)	0.0171 (14)
C9	0.0476 (17)	0.0497 (16)	0.0304 (12)	0.0046 (13)	0.0091 (12)	0.0103 (11)
C10	0.0514 (18)	0.0563 (18)	0.0382 (14)	0.0070 (14)	0.0151 (13)	0.0149 (13)
C11	0.061 (2)	0.0509 (17)	0.0470 (17)	−0.0009 (15)	0.0243 (16)	0.0027 (14)
C12	0.092 (3)	0.074 (2)	0.0359 (16)	0.000 (2)	0.0260 (19)	0.0160 (16)
C13	0.091 (3)	0.094 (3)	0.0468 (19)	0.026 (3)	0.023 (2)	0.037 (2)
C14	0.072 (2)	0.076 (2)	0.0431 (17)	0.0236 (19)	0.0166 (16)	0.0258 (16)
C15	0.064 (2)	0.068 (2)	0.0361 (14)	0.0342 (17)	0.0225 (14)	0.0267 (14)
C16	0.0503 (17)	0.0512 (16)	0.0351 (13)	0.0191 (13)	0.0156 (12)	0.0235 (12)
O17	0.0651 (15)	0.104 (2)	0.0362 (11)	0.0458 (15)	0.0230 (11)	0.0328 (12)
O18	0.0711 (17)	0.121 (2)	0.0421 (12)	0.0550 (17)	0.0221 (12)	0.0410 (14)
C19	0.096 (3)	0.0489 (18)	0.059 (2)	0.0244 (19)	0.032 (2)	0.0248 (16)

### Geometric parameters (Å, º)

**Table:**

Br1—C4	1.897 (3)	C8—H8B	0.9700
Cl1—C11	1.727 (4)	C9—C10	1.376 (5)
N1—C1A	1.365 (4)	C9—C14	1.380 (5)
N1—C7	1.378 (4)	C10—C11	1.379 (5)
N1—C8	1.458 (4)	C10—H10	0.9300
C1A—C2	1.384 (4)	C11—C12	1.384 (6)
C1A—C5A	1.411 (4)	C12—C13	1.340 (7)
C2—C3	1.364 (5)	C12—H12	0.9300
C2—H2	0.9300	C13—C14	1.379 (5)
C3—C4	1.391 (5)	C13—H13	0.9300
C3—H3	0.9300	C14—H14	0.9300
C4—C5	1.375 (5)	C15—C16	1.495 (4)
C5—C5A	1.390 (4)	C15—H15A	0.9700
C5—H5	0.9300	C15—H15B	0.9700
C5A—C6	1.425 (4)	C16—O17	1.210 (4)
C6—C7	1.370 (4)	C16—O18	1.291 (4)
C6—C15	1.485 (4)	O18—H18	0.8200
C7—C19	1.485 (4)	C19—H19A	0.9600
C8—C9	1.506 (4)	C19—H19B	0.9600
C8—H8A	0.9700	C19—H19C	0.9600
			
C1A—N1—C7	109.2 (2)	C10—C9—C8	120.8 (3)
C1A—N1—C8	123.7 (3)	C14—C9—C8	119.1 (3)
C7—N1—C8	126.7 (3)	C9—C10—C11	118.6 (3)
N1—C1A—C2	130.6 (3)	C9—C10—H10	120.7
N1—C1A—C5A	107.6 (2)	C11—C10—H10	120.7
C2—C1A—C5A	121.8 (3)	C10—C11—C12	121.0 (4)
C3—C2—C1A	118.1 (3)	C10—C11—Cl1	118.8 (3)
C3—C2—H2	121.0	C12—C11—Cl1	120.2 (3)
C1A—C2—H2	121.0	C13—C12—C11	119.7 (3)
C2—C3—C4	120.6 (3)	C13—C12—H12	120.1
C2—C3—H3	119.7	C11—C12—H12	120.1
C4—C3—H3	119.7	C12—C13—C14	120.6 (4)
C5—C4—C3	122.4 (3)	C12—C13—H13	119.7
C5—C4—Br1	118.3 (3)	C14—C13—H13	119.7
C3—C4—Br1	119.3 (2)	C13—C14—C9	119.9 (4)
C4—C5—C5A	117.8 (3)	C13—C14—H14	120.0
C4—C5—H5	121.1	C9—C14—H14	120.0
C5A—C5—H5	121.1	C6—C15—C16	117.1 (3)
C5—C5A—C1A	119.4 (3)	C6—C15—H15A	108.0
C5—C5A—C6	133.6 (3)	C16—C15—H15A	108.0
C1A—C5A—C6	107.0 (3)	C6—C15—H15B	108.0
C7—C6—C5A	106.9 (2)	C16—C15—H15B	108.0
C7—C6—C15	127.0 (3)	H15A—C15—H15B	107.3
C5A—C6—C15	125.8 (3)	O17—C16—O18	123.3 (3)
C6—C7—N1	109.2 (3)	O17—C16—C15	124.6 (3)
C6—C7—C19	129.3 (3)	O18—C16—C15	112.0 (3)
N1—C7—C19	121.4 (3)	C16—O18—H18	109.5
N1—C8—C9	112.4 (3)	C7—C19—H19A	109.5
N1—C8—H8A	109.1	C7—C19—H19B	109.5
C9—C8—H8A	109.1	H19A—C19—H19B	109.5
N1—C8—H8B	109.1	C7—C19—H19C	109.5
C9—C8—H8B	109.1	H19A—C19—H19C	109.5
H8A—C8—H8B	107.9	H19B—C19—H19C	109.5
C10—C9—C14	120.1 (3)		
			
C7—N1—C1A—C2	179.0 (3)	C15—C6—C7—C19	6.7 (5)
C8—N1—C1A—C2	5.5 (5)	C1A—N1—C7—C6	0.5 (3)
C7—N1—C1A—C5A	−0.5 (3)	C8—N1—C7—C6	173.8 (3)
C8—N1—C1A—C5A	−174.1 (3)	C1A—N1—C7—C19	178.9 (3)
N1—C1A—C2—C3	−179.9 (3)	C8—N1—C7—C19	−7.8 (5)
C5A—C1A—C2—C3	−0.4 (4)	C1A—N1—C8—C9	71.6 (4)
C1A—C2—C3—C4	−0.3 (5)	C7—N1—C8—C9	−100.8 (4)
C2—C3—C4—C5	0.5 (5)	N1—C8—C9—C10	51.7 (4)
C2—C3—C4—Br1	−178.6 (2)	N1—C8—C9—C14	−128.7 (3)
C3—C4—C5—C5A	0.1 (5)	C14—C9—C10—C11	1.6 (5)
Br1—C4—C5—C5A	179.2 (2)	C8—C9—C10—C11	−178.8 (3)
C4—C5—C5A—C1A	−0.8 (4)	C9—C10—C11—C12	−0.7 (5)
C4—C5—C5A—C6	179.5 (3)	C9—C10—C11—Cl1	178.7 (3)
N1—C1A—C5A—C5	−179.4 (2)	C10—C11—C12—C13	−0.5 (6)
C2—C1A—C5A—C5	1.0 (4)	Cl1—C11—C12—C13	−179.8 (3)
N1—C1A—C5A—C6	0.3 (3)	C11—C12—C13—C14	0.7 (7)
C2—C1A—C5A—C6	−179.2 (3)	C12—C13—C14—C9	0.1 (7)
C5—C5A—C6—C7	179.6 (3)	C10—C9—C14—C13	−1.3 (6)
C1A—C5A—C6—C7	−0.1 (3)	C8—C9—C14—C13	179.1 (4)
C5—C5A—C6—C15	−5.5 (5)	C7—C6—C15—C16	−109.5 (4)
C1A—C5A—C6—C15	174.9 (3)	C5A—C6—C15—C16	76.6 (4)
C5A—C6—C7—N1	−0.2 (3)	C6—C15—C16—O17	3.5 (5)
C15—C6—C7—N1	−175.1 (3)	C6—C15—C16—O18	−176.6 (3)
C5A—C6—C7—C19	−178.5 (3)		

### Hydrogen-bond geometry (Å, º)

**Table:**

*D*—H···*A*	*D*—H	H···*A*	*D*···*A*	*D*—H···*A*
O18—H18···O17^i^	0.82	1.87	2.679 (3)	170

Symmetry code: (i) −*x*+2, −*y*+1, −*z*+2.
